# Response to Letter to the Editor From He et al: “Dasiglucagon in Children With Congenital Hyperinsulinism up to 1 Year of Age: Results From a Randomized Clinical Trial”

**DOI:** 10.1210/clinem/dgaf102

**Published:** 2025-02-22

**Authors:** Diva D De Leon, Indraneel Banerjee, Sebastian Kummer, Sune Birch, Eva Bøge, Jelena Ivkovic, David M Kendall, Paul S Thornton

**Affiliations:** Congenital Hyperinsulinism Center, Division of Endocrinology and Diabetes, The Children's Hospital of Philadelphia, Philadelphia, PA 19104, USA; Department of Pediatrics, Perelman School of Medicine at the University of Pennsylvania, Philadelphia, PA 19104, USA; Department of Paediatric Endocrinology, Royal Manchester Children's Hospital, Manchester M13 9WL, UK; Department of General Paediatrics, Neonatology and Paediatric Cardiology, Medical Faculty and University Hospital Düsseldorf, Heinrich Heine University Düsseldorf, Düsseldorf 40225, Germany; Zealand Pharma A/S, Søborg 2860, Denmark; Zealand Pharma A/S, Søborg 2860, Denmark; Zealand Pharma A/S, Søborg 2860, Denmark; Zealand Pharma A/S, Søborg 2860, Denmark; Congenital Hyperinsulinism Center, Department of Endocrinology, Cook Children's Medical Center, Fort Worth, TX 76104, USA

To the Editor,

We thank He et al (1) for their positive feedback and appreciation of dasiglucagon's potential to make a substantial contribution to the management of infants with congenital hyperinsulinism (CHI). The main concern of He and colleagues is that the durations of the placebo-controlled and open-label treatment periods in the present study do not support a comprehensive efficacy assessment, and that they insufficiently reflect the long-term glycemic stability achieved with dasiglucagon (1). We agree with He et al that long-term data are important for a comprehensive assessment of dasiglucagon's safety and efficacy.

The present placebo-controlled study in infants with CHI up to age 1 year (NCT04172441) is part of a wider clinical development program for dasiglucagon in CHI. In addition to this study, the program also encompasses a previously published study in children aged 3 months to 12 years (NCT03777176, https://doi.org/10.1210/clinem/dgad648) as well as an open-label, long-term safety and efficacy study (NCT03941236), in which participants of the first two studies are enrolled. Therefore, the main aim of the present study was to assess dasiglucagon's efficacy in the youngest age group, while the long-term extension study will provide follow-up efficacy and safety data over several years. The data from the present study should thus be considered in the wider context of the entire clinical development program for dasiglucagon.

To assess the efficacy of dasiglucagon in the youngest age group, the present study was designed with a placebo-controlled treatment arm of 2 × 48 hours and a 21-day open-label period. Considering the short plasma half-life of dasiglucagon (2, 3) and its rapid onset of action (3, 4), the 48-hour treatment period was sufficient to allow for an adequate estimation of dasiglucagon's treatment effect, which was the main goal of this study. Furthermore, when considering the duration of the placebo-controlled treatment period, it is important to recognize that the participant population in this study was especially vulnerable to the effects of hypoglycemia, comprising infants who were dependent on intravenous (IV) dextrose to maintain euglycemia. Thus, the present study investigated the efficacy of dasiglucagon at an age requiring critical treatment decisions determining the life course of illness to prevent and manage disease-related complications manifested later in life. Therefore, during the study planning phase, ethical concerns were raised about an extended use of placebo in the study design, with the potential of lifelong harm from neuroglycopenia in the placebo arm if treatment was delayed. The chosen crossover 2 × 48-hour study design limited the exposure of patients at risk of severe brain injury to hypoglycemia, while enabling clear objective markers of dasiglucagon efficacy. The 21-day open-label period was mainly included in the study design to provide clinical benefit to all participants in this study beyond the placebo-controlled part of the study. Subsequently, all participants (apart from one) were enrolled in the ongoing long-term extension study.

Overall, the present study was designed to be able to observe a treatment effect with dasiglucagon, while at the same time limiting the exposure to placebo to protect the vulnerable participant population enrolled in this study. The observed reductions in IV glucose dependence and need for pancreatectomy are important markers of efficacy. We look forward to publishing the long-term efficacy and safety data in the near future.

## Clinical Trial Information

EU Clinical Trials registration number 2017-004545-24; ClinicalTrials.gov registration number NCT04172441.

## Abbreviations

CHI: congenital hyperinsulinism
IV: intravenous
